# Recent advances to enhance the immunomodulatory potential of mesenchymal stem cells

**DOI:** 10.3389/fimmu.2022.1010399

**Published:** 2022-09-23

**Authors:** Madina Sarsenova, Yevgeniy Kim, Kamila Raziyeva, Bexultan Kazybay, Vyacheslav Ogay, Arman Saparov

**Affiliations:** ^1^Department of Medicine, School of Medicine, Nazarbayev University, Nur-Sultan, Kazakhstan; ^2^Laboratory of Stem Cells, National Center for Biotechnology, Nur-Sultan, Kazakhstan

**Keywords:** mesenchymal stem cells, cell therapy, preconditioning, cytokines, 3D culturing, hypoxia, genetic modifications, immunomodulation

## Abstract

Considering the unique therapeutic potential of mesenchymal stem cells (MSCs), including their immunosuppressive and immunomodulatory properties as well as their ability to improve tissue regeneration, these cells have attracted the attention of scientists and clinicians for the treatment of different inflammatory and immune system mediated disorders. However, various clinical trials using MSCs for the therapeutic purpose are conflicting and differ from the results of promising preclinical studies. This inconsistency is caused by several factors such as poor migration and homing capacities, low survival rate, low level of proliferation and differentiation, and donor-dependent variation of the cells. Enhancement and retention of persistent therapeutic effects of the cells remain a challenge to overcome in MSC-based therapy. In this review, we summarized various approaches to enhance the clinical outcomes of MSC-based therapy as well as revised current and future perspectives for the creation of cellular products with improved potential for diverse clinical applications.

## 1 Introduction

MSCs are multipotent, non-hematopoietic stem cells that are found in many tissues and organs of adults, including bone marrow, adipose tissue, liver and muscle. They are characterized by fibroblasts-like morphology, expression of CD105, CD90, CD29, CD37 and lack of CD34, CD45, CD14, CD40, CD80, CD86 and HLA-DR. MSCs have low expression of class I and II MHC antigens, which explains their reduced ability to generate an immune response. They are widely distributed in the body and their functions and properties differ depending on tissue source. For example, MSCs from the bone marrow are preferably differentiated into osteoblasts, while adipose tissue-derived MSCs (AT-MSCs) differentiate into adipocytes. Overall, MSCs are predominantly differentiated toward osteoblast, adipocyte and chondrocyte lineages (1, 2). Under physiological conditions, MSCs reside in specific stem cell niches and are responsible for maintaining tissue homeostasis. Specifically, they are involved in normal cell turnover and the replenishment of adult cells after injury (3). For example, MSCs are a key component of the bone marrow stem cell niche where they provide physical support to hematopoietic stem progenitor cells (HSPCs) (4, 5). In particular, direct contact and paracrine activity of MSCs regulate development and differentiation of HSPCs.

MSCs are considered as a potential therapeutic target for regenerative medicine as they possess a number of unique features, which include high proliferative potential, ability to differentiate into multiple cell lineages, immunomodulatory properties as well as the ability to migrate and colonize injured tissues. Moreover, MSCs secrete paracrine factors responsible for the tissue remodeling and repair along with their ability to suppress the immune response (6). Thus, MSCs can be used in clinical applications for tissue regeneration, wound healing, drug delivery and immune system regulation (7–9). However, implementation of MSCs therapy into clinics is complicated by the fact that MSCs from different origins may behave differently. This functional heterogeneity depends on various factors, including the tissue of origin, the state of the donor, and the condition of the culture medium (10). Although previously it was considered that MSCs from different origins share similar characteristics, it is now widely accepted that MSCs express additional tissue-specific antigens on their surface that make them different from MSC colonies from other tissues and also determine their fate in differentiation and regeneration (10). Thus, a deeper understanding and evaluation of MSC heterogeneity may simplify and maximize the therapeutic effect of stem cell-based therapy.

Multiple studies have demonstrated that MSCs could promote repair and regeneration of cardiac, pulmonary, renal, hepatic, bone, cutaneous, and other tissues (11, 12). Earlier trials have hypothesized that MSCs mediated their therapeutic effects *via* migration to the damaged sites and differentiation into resident cells of the injured tissues. This suggestion had received some supporting data from several publications that reported homing to the damaged organs and differentiation of exogenous MSCs to epidermal basal cells, endothelial cells, Schwann cells, and possibly hepatocytes in rodent models (13–16). The recruitment and differentiation of MSCs were confirmed using fluorescently or luminescently labeled MSCs (for instance, green fluorescent protein expressing MSCs) or by quantitative PCR of human genes in the case when human MSCs were evaluated in animal models. Nonetheless, further investigations have found little evidence for the claim that MSCs exert their regenerative functions *via* cell replacement and differentiation. Thus, using the same methods of MSCs tracing with fluorescent markers, different research teams have demonstrated low engraftment of MSCs into heart, kidney, bone, retina, and liver in disease models of the aforementioned organs (17–19). Moreover, despite enhanced tissue repair and functioning after MSCs therapy, the studies have not found significant evidence for the differentiation of MSCs into other cell types. Therefore, the prevailing view now is that MSCs perform their therapeutic actions *via* paracrine mechanisms rather than by direct differentiation and cell replacement (20, 21). One of the paracrine effects of MSCs is modulation of the immune system.

MSCs mediate their immunomodulatory functions *via* mutual crosstalk with immune cells and the secretion of various mediators. An alteration of their interaction results in various pathologies, including cancer, metabolic disorders and hepatic failure (22). Furthermore, MSC homing and survival efficiency are the key components of successful cell therapy. MSCs can be administered either locally or systematically. When injected locally, i.e. directly into the damaged area, MSCs are navigated into the wound environment by pro-inflammatory factors expressed by immune and resident cells (6). When administered intravenously, MSCs circulate along blood vessels until they reach the required niche or injury site. Then they pass through the endothelial barrier and move toward the damaged tissue. The homing mechanism of MSCs includes chemotaxis, rolling, adhesion, diapedesis and interstitial migration (19). Exogenous MSCs possess infiltration features due to their abilities to adhere, migrate, and implant into the target site. After reaching the injured tissue, MSCs start to differentiate and secrete mediators, such as cytokines, chemokines and growth factors leading to tissue repair (13). Tissue-resident MSCs that are mobilized from their niches are activated by the damage-associated molecular patterns from dying cells and physical or chemical signals from the injured site (23).

In general, MSCs have three key features, namely plasticity, immunomodulation and self-renewal that make them an attractive tool for the use in regenerative medicine. This review article is focused on MSCs and their preconditioning strategies, such as cytokines and factors, immune receptor agonists, 3D culturing, hypoxia, autophagy, genetic modifications and other agents to improve MSC immunomodulatory potential for their further application in clinical practice. Before reviewing and discussing the main topic of this manuscript, the next section will briefly overview the immunomodulatory potentials of MSCs.

## 2 Immunomodulatory potential of MSCs

The immunomodulatory properties of MSCs are one of the key features that make them an attractive tool for cell therapy. MSCs can sense the injured site and induce an immune response in both innate and adaptive immunity in case of a low magnitude of the response. They also suppress the function of the immune cells if the damaged site is overactive (20). This “sensor and switcher of the immune system” function is regulated by various pathways.

According to the secretion level of factors in the inflammatory environment, MSCs display either pro-inflammatory or anti-inflammatory functions (24, 25). At low levels of IFN-γ and TNF-α, MSCs acquire the pro-inflammatory phenotype. In such conditions, MSCs produce chemokines and factors such as MIP-1α/β, RANTES, CXCL9, CXCL10, and CXCL-11 for further activation of T cells; PGE-2 for the disruption of DC precursors. Additionally, in the absence of IL-6, and the presence of IFN-γ and IL-1, MSCs promote proliferation and activation of M1 macrophages, which further express IFN-γ and TNF-α within the injured tissue. The anti-inflammatory function of MSCs lies in the immune response suppression in an environment with a high level of inflammatory cytokines production. Under high levels of IFN-γ and TNF-α, MSCs produce cytokines such as TGF-β, HGF, and secrete soluble factors such as IDO, PGE2 and NO. These factors directly promote the activation of regulatory T cells (Tregs) (CD4+, CD25+, forkhead box P3 (FOXP3+)). Furthermore, in response to IL-6 stimulation, MSCs secrete TGF-β and PGE2 again to induce Treg cell activation. Additionally, MSCs launch the expression of COX2 and IDO and further promote homeostatic response towards macrophage polarization. Macrophages with M2 phenotype express CD206 and CD163 co-stimulatory molecules, along with the expression of IL-6 and IL-10, which is produced by both DCs and M2 macrophages. This mechanism plays a role in boosting immunosuppression by suppressing effector T cells (26). Thus, the MSCs with anti-inflammatory phenotype restore the immune balance by the inhibition of T lymphocyte activation, and proliferation and activation of Tregs.

**Figure 1:**
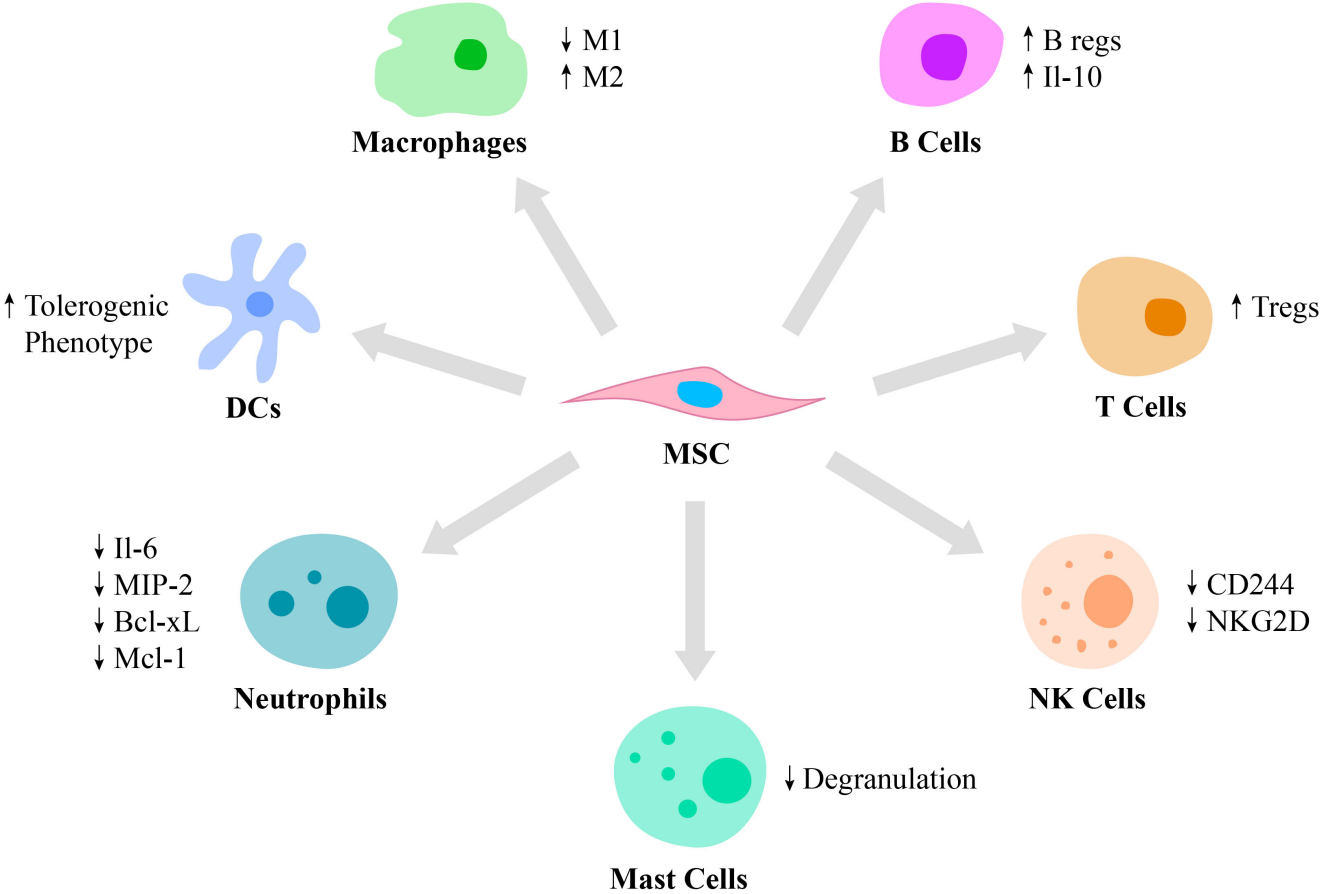
Immunomodulatory properties of MSCs. MSCs are capable of regulating the function of various cells of the immune system. Thus, MSCs stimulate macrophage polarization from the pro-inflammatory M1 phenotype to the pro-healing M2 phenotype and regulate DC differentiation toward the tolerogenic phenotype. Also, MSCs suppress mast cell degranulation and downregulate the production of IL-6, MIP-2, Bcl-xl and Mcl-1 by neutrophils and expression of CD244 and NKG2D by NK cells. Moreover, MSCs switch the phenotype of B and T cells toward Bregs and Tregs, respectively.

MSCs possess immunomodulatory functions primarily *via* the cell-to-cell contact with immune cells, such as monocytes, neutrophils, macrophages, dendritic cells (DCs), mast cells, natural killer (NK) cells, T cells and B cells (**Figure 1**). For example, they regulate an overactive immune response *via* interaction with specific anti-inflammatory immune cell phenotypes, such as Tregs and M2 macrophages (27). MSCs generate extracellular vesicles that enhance the proliferation of M2 macrophages and Tregs and at the same time, inhibit the activity of T and B lymphocytes as well as M1 macrophages (28). The polarization of macrophages toward M2 phenotype occurs due to transactivation of arginase-1 by signal transducer and activator of transcription 3 (STAT3) loaded into exosomes and secreted by MSCs (29, 30). Also, miR-182 secreted by MSCs directly mediates the polarization of macrophages from M1 to M2 phenotype (31).

Neutrophils are recruited by MSCs *via* secretion of IL-8 and macrophage migration inhibitory factor (2). MSCs regulate neutrophil apoptosis *via* the NF-κB signaling pathway. Incubation of activated human neutrophils in MSC cultured media resulted in reduced secretion of IL-6 and macrophage inflammatory protein 2, as well as decreased secretion of the anti-apoptotic molecules, Bcl-xL and Mcl-1 (32). Also, exosomes secreted by MSCs were reported to significantly enhance a respiratory burst of neutrophils in patients with severe congenital neutropenia (33).

MSCs were reported to suppress the immune response by modulating antigen-presentation by DCs (34). Moreover, MSCs regulate the differentiation of mature DCs toward a tolerogenic phenotype *via* the secretion of hepatocyte growth factor (HGF), which is mediated by activation of the HGF/Akt pathway (35). Furthermore, umbilical cord-derived MSCs (UC-MSCs) secrete FLT3L that binds to FLT3, which is expressed on peripheral tolerogenic CD1c+ DCs. This leads to CD1c+ DCs activation, proliferation and inhibition of their apoptosis (36).

In addition to neutrophils and DCs, MSCs also modulate mast cell functions in the inflammation area by reducing their degranulation (37). The role of mast cells in innate and adaptive immunity is critical, as they release mediators, including histamine, tryptase and IL-4. Activation and degranulation of mast cells accelerate the migration of lymphocytes to the injured site (38). However, hyperactivation of mast cells leads to various pathological conditions, including systemic anaphylaxis, which is a rapid immune reaction that in some cases causes death (39). Thus, MSCs suppression of mast cell degranulation is critical in some allergic conditions.

NK cells play an important role in several conditions, including cancer, tissue and organ transplantation, autoimmune disorders and other immune system-related diseases (40, 41). Therefore, MSCs and NK cells interaction is also critical to investigate. Thus, it was reported that MSCs play a dual role in NK cell function, by suppressing their cytokine production, proliferation and cytotoxic effect in some cases and stimulating their activation in others depending on the inflammatory milieu. In particular, studies report that MSCs downregulate activating receptors on NK cells, such as 2B4 (CD244) and natural killer group 2-member D (NKG2D), which are responsible for cytokine production and the cytotoxic function of NK cells (22).

In addition to innate immunity, MSCs also affect adaptive immunity by regulating B lymphocyte activation, proliferation, differentiation and apoptosis. MSCs switch B cell phenotype toward B regulatory cells (Bregs), which secrete IL-10, possess immunosuppressive functions and modulate the immune environment homeostasis (34). Also, IL-10 expressed by Bregs inhibits the production of inflammatory cytokines and proliferation of T lymphocytes (42). The ability of MSCs to suppress B lymphocyte proliferation and differentiation is partially regulated by the suppressor of cytokine signaling 1 (43). In addition, MSC-secreted extracellular vesicles contain mediators, such as Ezrin, Radixin, and Moesin (ERM) proteins and miRNA-155-5p, which are partially responsible for MSCs/B cells communication *via* a PI3K-AKT signaling pathway (44).

MSCs suppress T lymphocyte proliferation *via* cell-to-cell contact or through the secretion of soluble factors, such as IFN-γ and indoleamine 2,3-dioxygenase (IDO) (45). MSCs also secrete prostaglandin E2 (PGE2) that also inhibits T lymphocyte activation (43). Moreover, they switch the phenotype of pro-inflammatory T cells toward pro-healing Tregs by secreting transforming growth factor-β (TGF-β) (46). In addition to the cell-cell contact, MSCs regulate immune reactions by secreting soluble mediators such as cytokines, growth factors and other agents including PGE2, IDO and nitric oxide (47). Moreover, MSCs decrease the expression of pro-inflammatory molecules, such as tumor necrosis factor-α (TNF-α), IL-1, IL-6, IL-12p70, IFN-γ and enhance the production of anti-inflammatory mediators, including IL-10 (48). Some mediators, such as HGF or TNF-α-stimulated gene/protein 6 (TSG-6) expressed by MSCs, have been reported to be involved in the treatment of various immune system mediated diseases, such as multiple sclerosis (49). In particular, TSG-6 suppresses chemokine-stimulated transendothelial migration of neutrophils mediated by CXCL8 *via* antagonizing the binding of CXCL8 to heparin (50).

Various mediators that control MSC migration functions are also critical for successful therapy. It was demonstrated that the migration of MSCs is regulated by multiple factors, including stroma cell-derived factor-1 (SDF-1)/CXCR4 axis, osteopontin, basic fibroblast growth factor, vascular endothelial growth factor (VEGF), HGF, insulin-like growth factor-1, platelet-derived growth factor, TGF-β1 as well as the level of oxygen and mechanical signals (13). Among all, SDF-1 and CXCR4 are the main regulators of MSC migration. Thus, pretreatment of MSCs with SDF-1 in a diabetic mice model showed improved tissue repair in post-myocardial infarction heart (51). Similarly, CXCR4 stimulates MSCs migration toward the site of injury. The knockout of CXCR4 led to decreased MSC homing and survival (52). CCL2/monocyte chemoattractant protein-1 (MCP-1) and CCL3/macrophage inflammatory protein-1α (MIP-1α) were also reported to recruit MSCs to the damaged tissue. Thus, exogenously presented CCL2/MCP-1 and CCL3/MIP-1α greatly enhanced MSC homing efficiency, while silencing their expression decreased cell migration (53). In addition, the chemotaxis properties of CXCL12 can be enhanced by complement components, such as C1q, and bioactive lipids, including sphingosine-1 phosphate and ceramide-1 phosphate (2). Overall, immunomodulation is the key mechanism of action of MSCs, allowing the use of these cells in various therapies in the area of tissue and organ regeneration, immune system regulation, delivery of therapeutic agents and others.

## 3 Strategies to improve MSC potential

**Figure 2:**
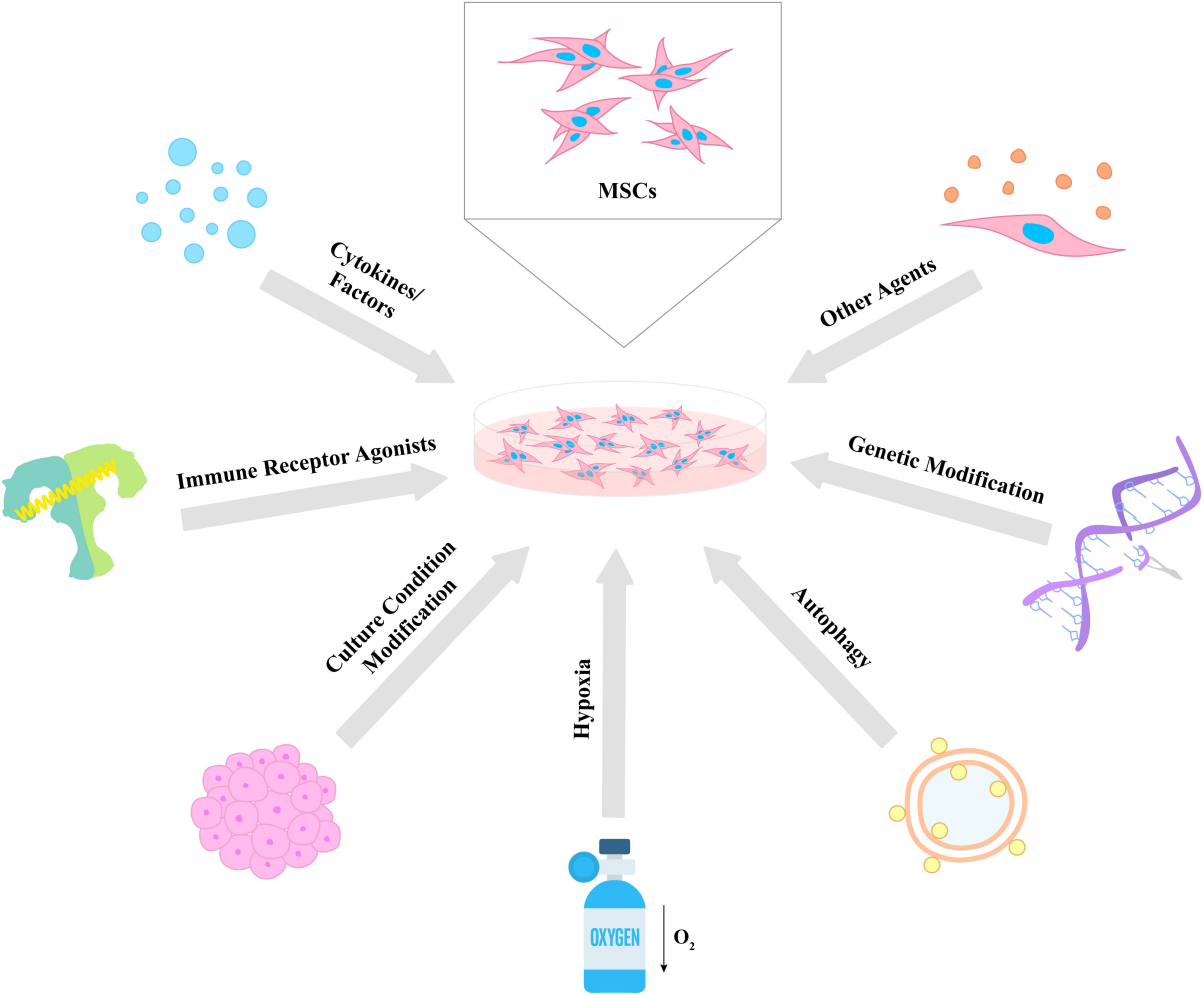
Different strategies for the enhancement of MSC therapeutic properties. Various approaches were used to enhance the immunomodulatory potential of MSCs including cytokines and factors, immune receptor agonists, culture condition modification, hypoxia, autophagy, genetic modification, and other agents. The proposed strategies lead to an increase in the survival, proliferative, secretory, homing, migratory, and differentiation capacities of MSCs, which results in the improvement of their therapeutic effects.

Considering the aforementioned immune regulatory properties of MSCs, scientists and clinicians have made many efforts in order to enhance and control the effects of the cells for future use in various disease treatments. In this chapter, we divide these strategies into seven categories: (i) cytokines and factors, (ii) immune receptor agonists, (iii) culture condition modification, (iv) hypoxia, (v) autophagy, (vi) genetic modifications, and (vii) other agents for the improvement of the therapeutic potential of MSCs (**Figure 2**).

### 3.1 Preconditioning with cytokines

The strategies of preconditioning MSCs with pro-inflammatory cytokines have proven to improve the therapeutic potential of MSCs by affecting their immunosuppressive properties including the production of anti-inflammatory molecules and increased homing to the site of injury (54–58).

#### 3.1.1 IFN-γ

IFN-γ is a pleiotropic immunomodulatory cytokine that affects both innate and adaptive immune responses by inducing inflammatory processes to protect organisms against various pathogens (59). Its production is generally restricted to activated lymphocytes including CD4+ Th1 cells, CD8+ T cells, γδ T cells, and NK cells (60). However, IFN-γ can also be secreted by B cells, DCs, macrophages, and monocytes (61). Early research studies have identified the critical role of this cytokine in activating and changing the phenotype of MSCs, and making it one of the most investigated cytokines used for MSC preconditioning.

At the molecular level, IFN-γ exposure leads to the phosphorylation of STAT1/3 and inhibition of ERK1/2-dependent mTOR signaling cascade in MSCs and activating pSTAT1 nuclear translocation, which further upregulates the immunoregulatory genes of MSCs (62). The study by Kim and colleagues demonstrated that IFN-γ induces JAK signal transducer and STAT1 signaling cascade, which leads to the production of IDOs by MSCs (63). However, it needs to be mentioned that sustained IFN-γ treatment for a longer period of time can inhibit STAT3, impairing MSC proliferation and differentiation, suggesting a need for further research and adjustments in its use.

Recent studies additionally showed that IFN-γ upregulates the expression of HLAI/II and their co-stimulatory proteins and increases the secretion of immunosuppressive molecules including IDO, TGF-β, PGE2, CCL2, and HGF (64). Such changes reduce MSCs’ susceptibility to NK cells. Preconditioned MSCs not only block IFN-γ production by NK cells *via* IDO and PGE2 but also protect themselves from NK cytotoxicity by increasing HLA-I and reducing NKG2D, the ligand required for NK cell activation (65). Additionally, secreted IDO has shown to inhibit T cell proliferation and CD8+ T cell degranulation suggesting enhanced protection from T cell cytotoxicity (66). It is important to note that upregulation of HLAI/II by MSCs in response to IFN-γ exposure may increase the immunogenicity of MSCs, making them susceptible to destruction by host immune cells following *in vivo* administration (67).

Wang and colleagues demonstrated that the IFN-γ priming upregulates genes of HLA-G5 and IDO as well as immunosuppressive factors in Wharton’s jelly MSCs, which is consistent with previous studies on MSCs from other tissue origins (68). This research revealed that IFN-γ preconditioning also blocks the release of IFN-γ and TNF-α, while enhancing the IL-6 and IL-10 production in MSCs. Preconditioning also helps with homing by increasing the expression of chemokine ligands CXCL9, CXCL10, and CXCL11 and adhesion proteins, vascular cell adhesion protein-1 (VCAM-1) and intercellular adhesion molecules-1 (ICAM-1). Additionally, the co-culturing of IFN-γ primed MSCs with lymphocytes leads to the inhibition of Th1 and Th17 proliferation with no effect on Th2 and Treg populations *in vitro*. All of these changes in MSC phenotype illustrate an improved immunosuppressive function of MSCs (65, 68). A similar effect was observed with AT-MSCs (69).

The experiments on animal models confirm the enhanced therapeutic potential of MSCs following the priming with IFN-γ. The administration of IFN-γ-pre-activated MSCs in rats with ischemia-reperfusion injury of kidneys induced by the renal artery ligation showed significantly decreased infiltration of pro-inflammatory immune cells to the site of injury reducing renal fibrosis (70). Interestingly, even the extracellular vesicles derived from IFN-γ-primed MSCs improved the fibrosis of the lung in mice with systemic sclerosis (71). Such an effect can be linked to the increased production of PGE2 by primed MSCs that expand the number of CD163+/CD206+ immunosuppressive macrophages in the site of injury resulting in reducing inflammation (70). Similarly, in the TNBS-induced mouse colitis model, pre-treatment with IFN-γ demonstrated a better migration rate of MSCs to the site of injury, reduced damage of mucosal tissue from inflammation, and overall increased survival rates (72). Since no serious adverse effects were detected in animal studies, currently, an open-label phase I clinical trial (NCT04328714) using IFN-γ-primed human bone marrow-derived MSCs (BM-MSCs) as graft versus host disease prophylaxis is being conducted in patients undergoing hematopoietic cell transplantation for the treatment of acute leukemia and myelodysplastic syndrome. Thus, these findings clearly confirm the importance of using IFN-γ for MSC activation to increase their therapeutic potential.

#### 3.1.2 TNF-α

TNF-α is a major pro-inflammatory cytokine found at the injury sites which is also known for diverse pleiotropic effects including regulation of cell growth, metabolism, and differentiation of various cell types. It is also a key regulator of the nuclear factor kappa light chain enhancer of activated B cells (NF-κB) signaling pathway controlling the expression of genes involved in cell survival, proliferation, differentiation and migration (73–75).

As MSCs express TNF-receptor (TNFR) 1 and 2, TNF-α/TNFR interaction can activate downstream IκB kinase proteins that release NF-κB by phosphorylating IκBα (76). It allows for the upregulation of NF-κB target genes including VCAM1, CD44 and matrix metalloproteinases (MMP9), and greatly increases the migration capacity of MSCs, while also stimulating MSC proliferation as it upregulates cyclin D1, the gene of the protein involved in the G1-phase of the cell cycle (74, 76, 77). Additionally, the TNF-α priming upregulates the expression of immunosuppressive factors IDO and PGE2, and increases the production of cytokines along with other factors including CXCL5, CXCL6, HGF, IL-8, insulin-like growth factor-1, and VEGF (78). According to Putra and colleagues, priming with TNF-α activates MSCs to start secreting high amounts of TGF-β and IL-10, resulting in the enhancement of their immunosuppressive phenotype (79).

This anti-inflammatory priming effect of TNF-α is also observed in and confirmed by animal experiments. The topical administration of TNF-α-activated MSCs to mice with experimental allergic conjunctivitis demonstrated a significant reduction in IL-4 and TNF-α levels and a drop in the number of inflammatory immune cells as well as NF-κB p65 expression *via* cyclooxygenase-2 (COX-2)-dependent mechanisms, which significantly improved the clinical outcome. The treatment also reduced the ability of mast and B cells to secrete IgE and release histamine, thereby preventing vascular hyperpermeability and blocking immune cells from reaching the site of inflammation (80). Similarly, the culture media and exosomes generated during MSC preconditioning with TNF-α also displayed an increased production of pigment epithelium-derived factor and VEGF-AA, growth factors, which showed the neuroprotective effect on retinal ganglion cells (81). These studies clearly demonstrate the potential of using TNF-α in MSC therapy. However, its effect is much less in contrast to IFN-γ priming (65). Therefore, currently, TNF-α is mostly used in combination with other cytokines.

#### 3.1.3 IL-1β

IL-1β is a key pro-inflammatory cytokine produced by various cell types including innate immune cells such as macrophages and monocytes, and very important in host defense against various pathogens (82). Therefore, it was intensely investigated for MSC priming. For instance, recent studies indicate that the IL-1β pretreatment significantly increases the immunomodulatory capacity of MSCs and enhances their migration to the site of inflammation. According to Liu and colleagues, IL-1β activates the NF-κB, TSG-6 and COX-2 pathways in MSCs, which explains their enhanced immunomodulatory ability (83). A different study confirmed these findings by showing that IL-1β induces COX-2-PGE2 signaling axis in MSCs that increased the secretion of PGE2, SDF-1, and VEGF. Additionally, they identified that IL-1β priming also blocks the MAPK-ERK1/2, PI3K-AKT, and NF-κB-P65 pathways in ischemia-reperfusion-injured tissue triggering local repair processes (84).

Interestingly, Yao and colleagues reported a separate mechanism in which IL-1β pretreatment upregulates the expression of miR-21 in MSCs. These microRNAs then get packaged into exosomes that are further released to transform pro-inflammatory macrophages into M2 anti-inflammatory macrophages. Both *in vitro* and *in vivo* experiments confirm these findings (85). This shows that IL-1β-preconditioned MSCs can affect the immune system in various ways and regulate inflammation successfully by using alternative mechanisms.

Furthermore, *in vivo* studies also illustrate IL-1β priming as a promising strategy. For instance, the intravenous infusion of IL-1β-primed MSCs stimulated the formation of Tregs in prostate, spleen, and lung tissue in mice with chronic prostatitis/chronic pelvic pain syndrome (CP/CPPS), disease characterized by chronic pelvic pain and prostatic inflammation. On the other hand, it also led to the inhibition of monocyte and pro-inflammatory macrophage infiltration significantly reducing their presence in the bloodstream, prostate, spleen, and lung. Priming with IL-1β also upregulated CXCR4 chemokine expression in MSCs, which assisted in their targeted migration. These resulted in the restoration of immunologic homeostasis, which further led to an alleviation of CP/CPPS symptoms (83).

Similarly, Aussel and colleagues investigated the immunomodulatory activity of IL-1β-preconditioned MSCs in rats with hemorrhagic shock-induced kidney and liver injury. The study revealed that the pretreated MSC injections reduced the levels of main organ injury markers including cystatin C, KIM-1, blood urea nitrogen and plasma creatinine. Moreover, the treatment reduced IL-1α, IL-6, IL-10 levels and downregulated the expression of CD80/86, PD-1/PDL-1 by granulocytes and monocytes (86). This indicates that the priming increased the therapeutic effect of MSCs on preventing organ injuries following hemorrhagic shock *in vivo*. Overall, these data demonstrate that priming with IL-1β can be used as an effective tool to activate MSCs and improve their anti-inflammatory and migratory characteristics.

#### 3.1.4 IL-17

IL-17 is a pro-inflammatory cytokine produced by various lymphocyte populations including CD4+ Th17, CD8+ Tc17, γδ T, NK cells, and ILC3s cells in response to extracellular pathogens or injury and it is involved in tissue inflammation by inducing the secretion of other pro-inflammatory cytokines and chemokines (87). Upon binding to its receptors, IL-17 stimulates the downstream signaling cascade, activating several pathways such as MAPK/AP-1 and NF-kB and influencing the expression of its target genes, which explains its role in the proliferation, differentiation and migration processes of immune cells (87).

At the molecular level, a study by Huang and colleagues investigated the role that IL-17 plays in the bone marrow microenvironment, the main place of contact with MSCs (88). According to their research, macrophages in the bone marrow naturally produce M-CSF when they directly contact Th17 cells that results in the production of IL-17. The cytokine then activates TRAF6/Act and Rac-dependent Nox1 protein, which increases the production of reactive oxygen species in MSCs residing in the bone marrow. This process is responsible for MEK/ERK hyperphosphorylation leading to MSC proliferation, migration and differentiation. Therefore, the use of IL-17 for priming MSCs can be considered as an artificial approach to mimic what happens naturally (88). As expected, the use of IL-17 for MSC preconditioning has demonstrated to effectively induce the proliferation of MSCs in a dose-dependent manner (65).

Unlike IFN-γ pre-activated MSCs, the use of IL-17 does not affect the expression level of both HLA I/II and their co-stimulatory factors, suggesting the maintenance of hypo-immunogenicity (89). It is advantageous considering the fact that MSCs are usually injected or transplanted. Although IL-17 treatment has no effect on the MSC morphology and does not inhibit the proliferation of MSC-co-cultured lymphocytes, it induces the secretion of high amounts of IL-8 and IL-6 and upregulates the expression of IDO, TLR3/4 and PD-L1 by MSCs, all of which are involved in promoting the anti-inflammatory function of MSCs (90). Importantly, IDO is one of the major immunomodulatory cytokines protecting MSCs from NK and T cell cytotoxicity as previously mentioned (65, 66) and TLR3/4 are danger sensing receptors activating MSC cytokine production. Despite the aforementioned beneficial effects of IL-17 on immunomodulatory capacity of MSCs, its further use requires additional research due to unexpected safety concerns. Thus, recent studies revealed that IL-17 treatment triggered accumulation of nitric oxide in MSCs upregulating the PD-L1 expression on their cell surface which might turn them into pro-tumorigenic MSCs (91) and stimulate their osteogenic differentiation (92), adding some limitations for their use.

Interestingly, Du-Rocher and colleagues showed that IL-17 enhances immunosuppressive effects of MSCs not directly but by bringing MSCs and lymphocytes into a closer contact. Specifically, IL-17 promotes the migration of MSCs to the site of inflammation by upregulating the expression of important migratory chemokines, CXCL8 and CCL8, and metalloproteases including MMP2 and MMP9 (90). Moreover, the preconditioned MSCs promote Tregs expansion over Th1 by blocking the production of Th1-related cytokines including IFN-γ, IL-2 and TNF-α. Surprisingly, increased IL-6 level in the supernatant of primed MSCs with T cells was significantly linked to the immunosuppressive effect rather than IDO, TGF-β or COX-2 (89). Thus, these data suggest that the priming of MSCs with IL-17 is an efficient strategy to increase the potency of the MSC therapy while maintaining low immunogenicity.

#### 3.1.5 Combination of cytokines

Pro-inflammatory cytokine cocktails are widely investigated as an additional strategy for priming MSCs for better therapeutic outcomes. For instance, even though MSC T cell suppressive function and secretion of immunomodulatory factors (PGE2, ICAM-1, CXCL16) differ depending on the types of cell lines and passages, the combination of TNF-α and IFN-γ was able to reduce this heterogeneity (93). According to Barrachina and colleagues (94), MSCs upregulate the expression of IDO, iNOS, IL-6, COX-2 and VCAM-1 upon combined TNF-α and IFN-γ treatment. It also promoted maintaining the differentiation potential and viability of MSCs (88). The administration of TNF-α/IFN-γ-primed murine MSCs in the equine model with osteoarthritis showed decreased synovial inflammation after two months. It increased COL2A1 (collagen type II), cartilage oligomeric protein, aggrecan, MMP2, and TGF-β1 and decreased COX-2 and IL-1β demonstrating improved therapeutic effect (95).

Additionally, the combined TNF-α and IFN-γ treatment increases the production of IDO that shifts monocyte differentiation towards immunosuppressive macrophages, which further increases the production of IL-10, thereby inhibiting CD3/CD28-induced T cell proliferation (78, 96, 97). Interestingly, TNF-α and IFN-γ treatment increases the secretion of extracellular vesicles by MSCs, which can inhibit T cell proliferation in a dose-dependent manner (98). These extracellular vesicles contain important anti-inflammatory factors including HGF, TSG-6, PGE2 and TGF-β, which induce macrophage polarization towards M2 phenotype and enhance Tregs number and activity in a mouse model of colitis (99).

Hackel and colleagues investigated the preconditioning of MSCs with TNF-α/IFN-γ in combination with IL-1β. The results demonstrated that this treatment enhances IFN-γR expression in MSCs, which is mediated by the NF-κβ signaling pathway. This makes MSCs more sensitive to IFN-γ, which further stimulates STAT5 and p38-MAPK signaling cascade and results in increased IL8 secretion and PMN recruitment (100). These findings suggest that MSC priming improved their ability to respond to lymphocytes that are recruited to the site of injury at later stages of inflammation, which might have an immunosuppressive effect (62, 100, 101).

Preconditioning of MSCs with IL-1β and IFN-γ was successful as well. It led to higher levels of nitric oxide, IL-6, and PGE2 release, regulating macrophage polarization and immunosuppressive effect (102). Combined IL-1β and IFN-γ treatment also inhibits the proliferation of peripheral blood mononuclear cells. The primed MSCs demonstrated upregulation of COX-2 and IDO mRNA expression compared to MSCs preconditioned by only one of the cytokines (103). The secreted PGE2/IDO inhibited Th1 differentiation, while promoting Tregs differentiation, showing positive clinical outcomes in the mouse model of colitis, clearly reflecting the therapeutic potential of using IL-1β/IFN-γ for priming MSCs. Overall, the preconditioning of MSCs with pro-inflammatory cytokines and their combinations has demonstrated the need to further investigate this strategy for improving MSC therapy. The use of various pro-inflammatory and immunomodulatory cytokines to enhance immunosuppressive qualities of MSCs in pre-clinical models of different diseases is summarized in **Table 1**.

**Table 1:** Preclinical studies of the effects of cytokine preconditioning on therapeutic and immunomodulatory properties of MSCs.

Cytokine	Type of MSCs	Preclinical Model	Therapeutic and Immunomodulatory Effects	Reference
INF-γ	Human AT-MSCs, BM- MSCs, CB-MSCs, and Wharton’s jelly MSCs	Mouse model of graft versus host disease	Increased IDO expression by MSCs	(63)
INF-γ	Rat BM-MSCs	Rat models of acute kidney injury and renal fibrosis	Decreased infiltration of pro-inflammatory immune cells to the site of injury reducing renal fibrosis and increased production of PGE2 by primed MSCs expanding the number of CD163+/CD206+ immunosuppressive macrophages in the site of injury resulting in reduced inflammation	(70)
TNF-α	Mouse BM-MSCs	Mouse model of allergic conjunctivitis	Lowered IL-4 and TNF-α levels, the number of inflammatory immune cells, and NF-κB p65 expression; significantly improved the clinical outcome	(80)
IL-1β	hUC-MSCs	Mouse – chronic prostatitis/chronic pelvic pain syndrome models	Activated the NF-κB, TSG-6, and COX-2 pathways in MSCs promoting their immunomodulatory actions	(83)
IL-1β	Human AT-MSCs	Mouse intestinal ischemia-reperfusion model	Induced COX-2-PGE2 signaling axis resulting in the increased secretion of PGE2, SDF-1, and VEGF; blocked the MAPK-ERK1/2, PI3K-AKT, and NF-κB-P65 pathways	(84)
IL-1β	Rat BM-MSCs	Rat hemorrhagic shock model	Reduced the levels of kidney injury markers including cystatin C, KIM-1, blood urea nitrogen, and plasma creatinine; decreased IL-1α, IL-6, IL-10 levels; and downregulated the expression of CD80/86 and PD-1/PDL-1 by granulocytes and monocytes	(86)
TNF-α + IFN-γ	Horse BM-MSCs	Equine model of osteoarthritis	Alleviated synovial inflammation; increased COL2A1, cartilage oligomeric protein, aggrecan, MMP2, and TGF-β1; and decreased COX-2 and IL-1β demonstrating improved therapeutic effect	(95)
IL-1β + IFN-γ	hUCB-MSCs	Mouse colitis model	Upregulated COX-2 and IDO expression; inhibited Th1 differentiation; promoted Tregs differentiation; and improved clinical outcomes	(103)

### 3.2 Immune receptor agonists

Another potential strategy for stimulating immunomodulatory functions of MSCs is treatment with immune receptor agonists. The most studied agents in this category are toll-like receptor agonists. Toll-like receptors (TLRs) are primary pathogen sensing type I transmembrane glycoproteins expressed mainly in immune cells. Due to their extracellular domain composed of rich leucine repeats, TLRs are able to recognize pathogen-associated molecular patterns and trigger intracellular adaptor molecules activating transcription factors including NF-kB, MAP kinases and IRF3/7 (104–106). The latter, in turn, induces the release of pro-inflammatory cytokines and other immune mediators contributing to protection against infection. TLRs are not only restricted to the cells of the immune system but also expressed on endothelial cells, epithelial cells, fibroblasts and MSCs as well. Therefore, the use of TLR agonists for MSC priming is considered as an alternative strategy to effectively activate the pro-inflammatory features of MSCs.

One of the TLRs, namely, TLR3 is highly expressed on MSCs and recognizes pathogenic double stranded RNA. Therefore, recent research has utilized synthetic analogs of TLR3 like polyribocytidylic acid [poly(I:C)] to activate MSCs (107, 108). TLR3-poly(I:C) ligation indeed promotes immunosuppressive properties of MSCs *via* the activation of the COX-2/PGE2 pathway (109), which, in turn, downregulates inflammatory cytokines (IFN-γ, IL-17A/-21/-23) and increases IL-10 production as well as inhibits Th1 and Th17 cell proliferation, while expanding the number of Tregs (110). Its therapeutic effects are also confirmed by an *in vivo* experiment on mice with TNBS-induced colitis where TLR3 activation of MSCs shifted Th1 response towards Th2. Additionally, it stimulated PGE2-dependent Treg induction leading to increased IL-10 production that resulted in the increased survival rate of mice (110). This effect can also be explained by the fact that the priming of TLR3 also increases the homing of MSCs to the inflammatory sites *in vivo* and enhances their efficacy (95). Moreover, Lim and colleagues demonstrated that this priming method can be further modified to improve the therapeutic potential of MSCs. The intraperitoneal injection of the IFN-γ and poly(I:C)-primed MSCs into the mice with colitis illustrated alleviated pathological condition of colon by decreasing the infiltration of immune cells into and the expression of pro-inflammatory cytokines in colon tissue, spleen and mesenteric lymph nodes and by promoting epithelial regeneration, enterocyte proliferation and Treg expansion (111). It also helped to restore the mucosal barrier. A similar effect is observed in the murine model for atopic dermatitis (112). All these findings demonstrate that poly(I:C)-priming indeed can improve the immunosuppressive properties of MSCs.

TLR4 is another well studied receptor found in many innate immune cells that recognize lipopolysaccharides (LPS) of invading microbes activating both innate and adaptive immune responses (113). Since TLR4 is expressed by MSCs, the use of LPS to stimulate TLR4-dependent intracellular signaling influencing the survival, proliferation, differentiation, migration and pro-inflammatory cytokine production by MSCs was investigated. The research by He and colleagues showed that TLR4 activation by LPS led to enhanced proliferation and differentiation of MSCs and promoted the production of IL-6 and IL-1β *in vitro via* Wnt3a/5a signaling (114). These results are confirmed by the changes in MSCs’ transcriptome which demonstrated increased cytokine production (100), enhancing the recruitment of neutrophils and macrophages to the wounded site and inducing NET formation. Both processes accelerate wound healing while reducing inflammation, clearly showing the efficacy of using TLR4-LPS ligation for MSC activation (115).

Supposedly, cytokines produced by MSCs in response to TLR4 stimulation are secreted as enclosed units bound within the exosomes. Kink and colleagues demonstrated, for instance, that the exosomes derived from LPS-treated MSCs are able to activate macrophages in a dose-dependent manner triggering their phagocytic function while promoting tissue repair (116).

Interestingly, Giallongo and colleagues showed that multiple myeloma cells could also activate TLR4 on MSCs inducing pro-tumor phenotype and supporting an immunosuppressive microenvironment (117). On top of that, LPS, depending on the time of exposure, may change the MSC phenotype from pro-inflammatory to immunosuppressive and vice versa which might put some limitations on its application (118). Nevertheless, these findings suggest that TLR4-LPS ligation induces Tregs to the site of injury and thus can be used to manage inflammation.

It is important to note that several TLRs agonists can be synergistically used to boost immunomodulatory qualities of MSCs. Thus, Rashedi and colleagues discovered that combined preconditioning of MSCs by TLR3 and TLR4 agonists, poly(I:C) and LPS, respectively, led to the enhanced proliferation of Tregs, increased expression of IL-10 and TGF-β1 and reduced IFN-γ and TNF-α expression *in vitro* (107). The priming with TLR agonists also upregulated DL-1 expression on MSCs and Notch1 with HEY1 expression suggesting the involvement of the Notch signaling pathway and its ligand, DL1, in the promotion of Treg differentiation by activated MSCs. Both the silencing of TLR3/4 genes using siRNAs and blocking Notch signaling using γ–secretase inhibitor DAPT confirmed the role of TLRs and the Notch pathway in inducing the differentiation of naïve T cells into Tregs (107). Therefore, the combination of TLR3/4 can be applied as an effective tool to improve immunomodulation of MSCs by promoting Treg differentiation and the increased secretion of immunomodulatory molecules (107, 119, 120).

Flagellin, a component of the flagellum mainly found in Gram-negative bacteria, is another immune receptor agonist that can be utilized to enhance MSC therapeutic properties. Specifically, flagellin activates TLR5 receptors which are expressed on MSCs (121). The study by Linard and colleagues demonstrated that flagellin-pre-treated BM-MSCs overexpressed IL-10 and promoted Treg proliferation while inhibiting Th17 differentiation (122). Other studies reported enhanced immunosuppressive attributes of MSCs treated with flagellin - upregulation of COX-2, shifting macrophage polarization towards M2 phenotype, and increased anti-inflammatory effects (123). Nevertheless, flagellin actions on MSCs should be further investigated since some studies showed that MSCs isolated from the umbilical cord (UC) when treated with flagellin induce the production of pro-inflammatory cytokines including IFN-β, IL-1β, IL-6, IL-8, IL-12, TNF-α, NF-κB and inhibits IL-10, CCL5, IP10 (124). It affects neither the proliferation of PMBCs nor the differentiation of UC-MSCs. Overall, priming MSCs with immune receptor agonists like TLR ligands holds great potential for the enhancement of their immunomodulatory capacity. The effects of TLRs on the immunoregulatory properties of MSCs in various animal models of diseases are outlined in **Table 2**.

**Table 2:** Preclinical studies on the effects of immune receptor agonists preconditioning on therapeutic and immunomodulatory properties of mesenchymal stem cells.

Immune receptor agonist	Type of MSCs	Preclinical Model	Therapeutic and Immunomodulatory Effects	Reference
Poly(I:C), TLR3 ligand	human UC-MSCs	Mouse model of colitis	Downregulated inflammatory cytokines (IFN-γ, IL-17A/-21/-23); increased IL-10 quantities; reduced Th1 and Th17 cell proliferation; expanded the number of Tregs; and improved survival of diseased animals	(110)
Poly(I:C), TLR3 ligand and IFN-γ	Mouse BM-MSCs	Mouse model of colitis	Decreased the infiltration of immune cells into and the expression of pro-inflammatory cytokines in colon tissue, spleen and mesenteric lymph nodes; promoted epithelial regeneration, enterocyte proliferation and Treg expansion; and restored the mucosal barrier	(111)
Poly(I:C), TLR3 ligand	Human Wharton’s jelly MSCs	Mouse model of atopic dermatitis	Reduced immune infiltration into and expression of pro-inflammatory cytokines in the skin	(112)
Flagellin, TLR5 ligand	Rat BM-MSCs	Rat radiation-induced proctitis model	Stimulated IL-10 expression and promoted Treg proliferation while inhibiting Th17 differentiation	(122)

### 3.3 Culture condition modification

In addition to cytokine and immune receptor agonist preconditioning, modifications in culture condition can serve as an alternative strategy for preconditioning (125). Even changes at the confluence of MSCs in culture can upregulate the expression of genes related to proliferation, homing and immunomodulation. It was demonstrated that cells at high confluency express genes responsible for homing, migration, differentiation, immunomodulation and angiogenesis, while low confluency cells showed an increased level of genes that are responsible for proliferation (126). The results can be explained by the upregulation of the chemokine ligand coding genes such as CCL2, CCL8, CXCL1, CXCL2, CXCL5, CXCL6, CXCL8 and CXCL16 at a high confluency of MSCs. Additionally, the expression of IL-1β and IL-6, which are one of the major mediators in inflammation, was also observed. These genes participate in the angiogenesis activation, cell survival rate, proliferation and chemotaxis of endothelial cells and T cells *via* autocrine and paracrine signaling mechanisms.

Similarly, the modification in cell culture such as three-dimensional (3D) condition improves sustainable maintenance of the stemness, homing and migration, and the ability to modulate the immune system by MSCs (65, 125, 127). The 3D culture provides a microenvironment that is close to the native one and mimics physiologically relevant conditions (128). Additionally, during ex vivo 2D conventional cell growth, cell‐surface molecules might decline in activity causing the dysfunction in cell‐cell adhesion. In this regard, 3D spheroid cultures were reported to promote the expression of surface molecules responsible for cell adhesion and survival. On the other hand, 3D culturing of aggregates generally leads to insufficient nutrients and oxygen supply. And, as a result, it creates an ischemic-like microenvironment suggesting the induction of hypoxic condition, which also enhances the immunomodulatory capacities of the stem cells (129).

Researchers have made many efforts in 3D culture priming approaches. Among these methods, the simplest approach is generating and maintaining a culture of a 3D spheroid that promotes the stabilization of HIF-1α, which is an important factor in a hypoxic environment (129). Sun and colleagues compared the therapeutic effects of 3D UC-MSC culture to the conventional 2D cell condition (130). Intravenous injection of 3D UC-MSCs reduced the plasma transaminase levels and pathological scores in severe ischemia-reperfusion injury model, whereas *in vitro* data on RNA sequencing demonstrated that 3D culture upregulated the expression of both pro-inflammatory and anti-inflammatory genes at the transcriptional level compared to the 2D cultured cells. However, authors also revealed the high expression level of ZC3H12A protein that is responsible for RNase encoding, which, in turn, breaks down the mRNAs of IL2, IL6, CXCL1, CXCL2 and CXCL3. The results of the study possibly indicates a new mechanism for the increased anti-inflammatory properties of 3D preconditioned MSCs, which is mediated at the post-transcriptional level. Additionally, in another recent study, researchers proposed an approach for the enhancement of MSC properties by culturing cells in 3D spheroids in combination with additional hypoxia exposure which was provided by hypoxia-mimetic small chemical compound, dimethyloxalylglycine (131). 3D-cultured MSC spheroids under hypoxic-like conditions exhibited the increased survival rates and expression levels of immunoregulatory factors such as TSG-6, MMP-2, and VEGF as well as improved homing and adhesion properties to injured tissue by the expression of CXCR-4.

Thus, compared to other 3D culture priming strategies, the 3D spheroid technique offers some advantages such as relative simplicity in generation, no need for matrix support and other agent supplementation and consequently, a lower cost. At the same time, 3D constructs for MSCs preconditioning have to provide and maintain important cellular functions. In this regard, scaffolds such as hydrogels might be an appropriate constructive supporting material to facilitate cytocompatibility (132, 133). For instance, Falcones and colleagues proposed a strategy for lung MSC preconditioning by expanding them in bioink from decellularized porcine lung which promoted the environment to the cells very similar to native extracellular matrix (ECM). This approach improved cell adhesion capacity, the length of the focal adhesions as well as decreased the secretion of pro-inflammatory cytokines. Moreover, 3D culture modification increased the expression of the CXCR4 receptor more than 20-fold, suggesting that the regulation of SDF-1-CXCR4 pathway might be altered when cells cultured under conventional condition (134). However, single 3D culture priming cannot be sufficient for increasing the immunoregulatory capacities of MSCs. A possible strategy to improve MSC properties is a combination of 3D culture with supplementary agents. Thus, the ECM scaffold coated with citric acid together with physical impact such as an alternating magnetic field significantly enhanced chondrogenic differentiation in MSCs by upregulation of chondrogenesis-related genes, COL2A1 and ACAN (135). In addition, pharmacological agents in combination with chitosan hydrogel similarly have positive effects on cell viability and proangiogenic paracrine activity. Touani and colleagues demonstrated that the combination of MSC pretreatment with celastrol and encapsulation in hydrogel improved therapeutic effects of the cells through the activation of proliferation, increase of anti-inflammatory factor release and endothelial cell proliferation (136). In conclusion, these studies have revealed that modifications in culture microenvironment efficiently impact the MSC potential associated with homing, survival, proliferation and differentiation, leading to the enhancement of their anti-inflammatory and immunomodulatory functions.

### 3.4 Hypoxia preconditioning

Hypoxia preconditioning of MSCs showed promising results in improving their immunoregulatory functions. MSCs are usually maintained *in vitro* under normal oxygen concentration. However, harsh hypoxic conditions at the site of tissue injury tend to inhibit MSCs survival and lead to their apoptosis (137). Pre-treatment of MSCs with hypoxia, on the other hand, can prepare the cells for the stern conditions at the transplantation site (65). Hypoxic treatment leads to the activation of anti-apoptotic signaling pathways by the induction of proangiogenic factors (138). The cellular response to hypoxia is generally controlled by the transcription factor hypoxia-inducible factor (HIF)-1, which controls numerous cellular processes including the metabolism (favoring anaerobic glycolysis over aerobic oxidative metabolism), angiogenesis and erythropoiesis (139). For instance, HIF-1α, a member of the HIF-1 family located in the cell nucleus, mediates the expression of regeneration-promoting genes such as VEGF (140). Furthermore, hypoxia induces the secretion of SDF-1α or CXCL12, a potent chemotactic factor for MSCs and other cells. Several other anti-inflammatory and immunoregulatory enzymes and cytokines are also upregulated by hypoxia pre-exposure such as IDO, IL-10 and IFN-γ (137, 141, 142). The aforementioned benefits of hypoxia preconditioning are exemplified by a recent study where low oxygen conditions at 1% level resulted in protection of cord blood MSCs by the activation of the anti-apoptotic mechanisms and strengthened their potential for pro-angiogenic factors production through VEGF expression (143). Similarly, hypoxia-preconditioned MSCs showed significantly lower expression levels of apoptotic and inflammatory proteins, and an upregulation of GLUT-1, GLUT-2 and GLUT-3 glucose transporters (144). This finding suggests that high glucose uptake in hypoxia-primed MSCs is involved in the promotion of survival and proliferation of these cells at the injury site. Another protein involved in MSCs survival, proliferation and augmentation of angiogenic cytokine production is glucose-regulated protein (GRP78) that promotes the potential of the AT-MSCs through the HIF-1α-GRP78-Akt signal axis (145). Additionally, MSCs preconditioned with hypoxia were shown to acquire anti-oxidant properties, which was demonstrated by lower levels of reactive oxygen species (146).

Interestingly, hypoxia exposure in combination with other agents has an amplifying effect on MSCs by enhancing the mechanisms of action for anti-apoptosis, survival, proliferation and angiogenesis. Thus, the expression pattern of numerous immunosuppressive and immunomodulatory proteins such as IDO, PD-L1, HLA-E, HLA-G in preconditioned MSCs can be increased by IFN-γ priming, while hypoxia simultaneously launches MSCs glycolysis leading to an increase in lactate levels, which is able to participate in T cell inhibition (147). These findings indicate that IFN-γ priming together with hypoxic stress provide synergistic effects on MSC capacities, and the combination of these factors is substantial for gaining MSC immune regulatory phenotype (148). Similarly, hypoxic preconditioning of MSCs before the generation of 3D spheroids enhances cell viability, proangiogenic potential of epithelial cells, and leads to bone tissue regeneration through the activation of HIF-1α (149).

Up until recently, studies on MSCs were limited by investigations of the cells only, however preconditioning of MCS secretome, including exosomes, might be a tool for therapeutic proposes as well. Several studies showed that exosomes from hypoxic conditions exhibited increased angiogenesis capacity appearing to be functionally more potent than normoxic MSC-derived exosomes (150, 151). Interesting data by Wang and colleagues demonstrated that priming with IFN-γ in combination with hypoxic condition of induced pluripotent stem cells derived MSC secretome induced higher pro-angiogenic gene and protein expression in endothelial cells. MSC secretome stimulated upregulation of IL-8 and VCAM-1 gene expressions in treated HUVECs (150).

The expression pattern of exosomal miRNA also provides the therapeutic potential of preconditioned exosomes. In this context, hypoxia preconditioning induced exosomal miR-126 production through the activation of HIF-1α in exosomes (152). Similarly, miR-612 was revealed as a functional messenger that stimulates angiogenesis through HIF-1α-VEGF signaling in endothelial cells (153). These findings suggest that preconditioning of MSC exosomes lead to the overexpression of miRNA content, including miR-126 and miR-612, and may represent a promising strategy for improving angiogenesis.

In conclusion, in accordance with the aforementioned studies, MSCs preconditioned with hypoxia alone or in combination with other factors as well preconditioned MSC exosomes have greater immune balancing potency than normal MSCs. Thus, the effects of hypoxic regulation of MSCs should be considered as a promising strategy for the clinical applications.

### 3.5 Autophagy modulation

Autophagy is a vital cellular process by which organelles and molecules are transported to the lysosome, degraded and recycled (154). It has homeostatic and cytoprotective functions, namely, it provides nutrients and energy in response to various stress types and during development as well as removes damaged cellular components, harmful substances and intracellular pathogens (155). Depending on the way the cargo is delivered to the lysosome, autophagy is classified into three types – macroautophagy, microautophagy and chaperone-induced autophagy (156). During macroautophagy, cytosolic components reach the lysosome in a double-membrane vesicle termed autophagosome. Macroautophagy is frequently used interchangeably with autophagy. We will use the latter term to substitute the former throughout the remainder of this article. Besides its fundamental functions in cell homeostasis, the latest research suggests that autophagy also controls the immunomodulatory actions of MSCs (157).

Several studies have reported that stimulation of autophagy could enhance the immunomodulatory properties of MSCs for the treatment of various diseases. A recent study demonstrated the beneficial effects of autophagy amplification in MSCs in a murine model of inflammatory bowel disease (158). Specifically, the researchers tested a novel autophagy enhancer called PACER for its immunosuppressive functions and found that the protein is crucial for immunomodulation by MSCs. Thus, knock-down of the Pacer gene using siRNA resulted in decreased levels of prostaglandin endoperoxide synthase 2 enzyme (PTGS, a principal regulator of immunomodulatory functions of MSCs) and poorer suppression of T cell proliferation in MSCs culture. On the contrary, the overexpression of the gene was associated with higher levels of PTGS and more efficient suppression of T cell division compared to normal MSCs. These positive findings were achieved in dextran sulfate sodium-induced mouse model of colitis. That is to say that MSCs-overexpressing PACER reduced inflammation in the colon of diseased animals and significantly improved the symptoms of colitis. One mechanism by which autophagy could boost immunomodulatory capacity is through an increase in Tregs and suppression of Th1 cells and inflammatory cytokines. A study by Cen and colleagues recently found that induction of autophagy in MSCs using rapamycin enhanced CD4+ T cells recruitment, promoted higher numbers of Tregs and decreased Th1 polarization and levels of inflammatory cytokines IL-17A, IFN-γ and IL-2 (159). By contrast, these effects were reversed when human bone marrow MSCs were treated with autophagy inhibitor 3-methyladenine. Similarly, Rossi and colleagues discovered that autophagy stimulator tamoxifen suppressed CD4+ T cells proliferation and activation in BM-MSC precursor cells, namely, marrow-isolated adult multi-lineage inducible (MIAMI) cells (160). Interestingly, chloroquine, which is known as a classic inhibitor of autophagy, mediated the same effects on MIAMI cells.

Inhibition of autophagy has also been demonstrated to promote the immunosuppressive functions of MSCs, as was mentioned above. According to a recent study by Wang and colleagues, autophagy in MSCs is responsible for their weakened immunosuppressive and anti-fibrotic potential when these cells are applied to treat hepatic fibrosis (161). The investigators showed that a fibrotic liver pro-inflammatory environment characterized by high levels of TNF‐α, IFN‐γ and TGF‐β1 activated autophagy in MSCs *in vitro* and in a mouse model. Specifically, incubation of MSCs with the three aforementioned factors resulted in increased formation of the autophagosome. Similarly, in CCl4-induced murine models of liver fibrosis, transplanted MSCs had elevated levels of an important autophagy marker MAP1LC3, which was not the case in the control group. Furthermore, it was found that autophagy was stimulated in MSCs *via* upregulation of autophagy-related gene Becn1. The authors then proposed that immunosuppressive and anti-fibrotic properties of MSCs could be recovered by suppressing autophagy *via* Becn1 silencing. This was successfully accomplished as treatment with Becn1-knockdown MSCs was associated with increased concentrations of PGE2 – one of the immunosuppressive mediators secreted by MSCs. In addition, inhibition of autophagy in MSCs resulted in decreased infiltration of CD4+ and CD8+ T lymphocytes in the fibrotic liver. Among other positive effects of Becn1 silencing was attenuation of fibrosis that was demonstrated by a smaller fibrotic area and reduced collagen deposition in hepatic tissue. Thus, the findings suggest that autophagy inhibition *via* Becn1 gene knockdown holds a potential to enhance immunomodulatory and other therapeutic effects of MSCs for the treatment of hepatic fibrosis. Similar results were reported by Tian and colleagues when silencing of the Becn1 gene has been linked with improved immunomodulatory properties of MSCs (162). In this study, the researchers discovered that knockdown of Becn1 using siRNA was associated with down-regulation of Th17 but upregulation of Tregs by MSCs in a mouse model of colitis subjected to restraint stress. Moreover, higher expression of TNFAIP6/TSG-6 (tumor necrosis factor, alpha-induced protein 6/TSG-6) and PGE2 were observed in response to autophagy inhibition by Becn1 silencing. The two proteins TNFAIP6/TSG-6 and PGE2 were previously reported to stimulate Treg production by MSCs (163, 164). These results confirm the beneficial effects of autophagy inhibition on immunosuppressive actions of MSCs. Another interesting finding in the study by Tian and colleagues is that activation of autophagy is one of the causes of reduced MSCs therapeutic efficiency in colitis with psychological stress. To be specific, stress led to an increased concentration of exosomes containing miRNA 7k and the latter inhibited STAT3 signaling, which in turn induced Becn1 expression and stimulation of autophagy. Accordingly, when researchers blocked miRNA 7k, autophagy was inhibited and the immunosuppressive effects of MSCs, such as the expression of anti-inflammatory PGE2, were recovered. All in all, both autophagy activation and suppression exert beneficial effects on T cell immunomodulatory effects *via* similar mechanisms, which include stimulation of Tregs and down-regulation of Th1 cells and pro-inflammatory cytokines. This enhancement of immunoregulatory effects were successfully utilized in animal models with a number of conditions such as inflammatory bowel disease, liver fibrosis, ischemic stroke and others.

### 3.6 Genetic manipulations

Another possible strategy to improve the therapeutic properties of MSCs is genetic modification. Genetic modification is targeted to enhance cell migration, homing, adhesion and survival in an inflammation area, as well as to maintain cell proliferation and differentiation, and to avoid senescence (165). The principle of the MSCs genetic modification is based on the viral vector with a recombinant DNA coding the gene of interest. The gene cassette carried by the vector is further loaded into the cell where it can be replicated or overexpressed. Transgene expression can either be constant with a continuous synthesis of specific molecular proteins, or be regulated by a gene switch (166).

Vectors inside the cell act as the inducers of specific genes that are responsible for migration, homing, adhesion, survival and proliferation. Concerning strengthening of the MSC migratory and homing abilities, the CXC chemokine receptors 1, 4 and 7 overexpression was utilized (166, 167). Hence, CXC chemokine receptor 7 (CXCR7) has been identified as a receptor of SDF-1 involved in MSC migration. The study by Shao and colleagues demonstrated the therapeutic effects of rat bone-marrow derived MSCs overexpressing CXCR7 (MSCs-CXCR7) in an acute lung injury (ALI) model (168). MSCs-CXCR7 administered into the ALI rats significantly improved homing and migratory abilities of the cells. Moreover, CXCR7 overexpression facilitated MSCs differentiation into type II alveolar epithelial cells, which additionally increased the anti-inflammatory capacity of MSCs by inhibition of TNF-α, IL-6 and IL-1β level expression. Additionally, recently it has been investigated that α(1,3) fucosyltransferse FUT7 enzyme improves cellular homing by binding E-selectins, which are known as cell adhesion molecules. FUT7 overexpression combined with non-covalent coupling of a recombinant PSGL-1 variant, 19Fc[FUT7+], targeted for P-selectin engagement, resulted in better homing, migration and adhesion of the functionalized swine bone-marrow MSCs with endothelial cells (169). Therefore, specific gene overexpression combined with the cell surface glycoengineering can be an alternative tool for MSCs therapeutic properties enhancement.

The factor in which researchers are interested in for the enhancement of the MSC abilities is cell survival because the existing strategies for MSC application without any modifications are restricted by low cell survival and engraftment. In this regard, overexpression of integrin-linked kinase (ILK) led to a significant increase in the expression and secretion of IL-6 by MSCs under hypoxic stimulation. Moreover, the survival and self-renewal of MSCs exposed to hypoxia were enhanced after ILK overexpression. The possible mechanism is in the increased levels of IL-6 that switched the JAK2/STAT3 signaling, and an lncRNA involved in Wnt pathway activation that is crucial for MSCs viability (170). Additionally, the overexpression of Gremlin1 protein implicated in cell growth, differentiation and development showed promising results in the enhancement of the MSC and endothelial cell survival in the ischemic hindlimb mouse model (171). Similarly, MSCs overexpressing VEGF and Bcl-2 also demonstrated evidence of improvement in MSCs capacities for further therapeutic application. Dual genetic modification resulted in a higher expression level of the target genes, a more rapid proliferation, reduced apoptosis, decreased autophagy and an enhanced paracrine effect (172). Another gene of interest for the enhancement of the MSC therapeutic capacities is heme oxygenase‐1 (HO‐1). HO-1 is a stress‐inducible enzyme that can catalyze the pro‐oxidant heme into biliverdin, CO and free‐iron, exerting powerful antioxidant effects. Liu and colleagues constructed genetically modified bone marrow derived MSCs (BM-MSCs) overexpressing HO‐1. While using ischemia-reperfusion induced acute kidney injury (AKI) kidney homogenate supernatant mimicking the AKI microenvironment, the authors found that HO‐1‐BM-MSCs showed an improved survival rate. The protective mechanism was due to the antioxidant, anti‐apoptosis and anti‐inflammatory effects of HO‐1 overexpression and through the activation of PI3K/Akt and MEK/ERK pathways. This genetic manipulation resulted in enhanced survival rate of MSCs in AKI (173).

Gene modification individually is a promising and advanced strategy for preconditioning; however, this approach can be strengthened by a combination with other agents. In this regard, Lee and colleagues suggested culturing of AT-MSCs in 3D condition for accelerated gene transduction (174).

Apart from the genetic modification of MSCs based on the vector with a recombinant DNA coding the target gene, there is a cutting-edge technology that has attracted the attention of scientists. Clustered regularly interspaced short palindromic repeats (CRISPR) and CRISPR-associated protein 9 (CRISPR-Cas9) together is the technology that allows a genetic sequence to be added, removed or altered at particular locations in the genome. In the context of MSCs modification, genome editing technology allows for enhanced survival and differentiation levels of the cells as well as an increase in the rate of proliferation in wound healing *in vivo* (175). In another study, manipulations with the Keap1 gene in AT-MSCs increased the anti-oxidative capacities with further increase of cell viability after grafting (176). Thus, genetic manipulations of MSCs aim to improve the capacities of the cells to exert an improved therapeutic efficacy. However, the therapeutic benefits need to be investigated by further clinical studies.

### 3.7 Other agents

In this section, we will discuss miscellaneous compounds and strategies used to improve the immunomodulatory potential of MSCs. One group of such agents are medications that are approved for the treatment of various health conditions. Interestingly, several of them have been shown to boost the immunomodulatory properties of MSCs. For instance, Deng and colleagues identified, for the first time, chlorzoxazone, an FDA-approved skeletal muscle relaxant, as an inducer of anti-inflammatory phenotype and enhancer of immunosuppressive actions of MSCs both *in vitro* and in a rat model of acute kidney injury (AKI) (177). Chlorzoxazone (CZ) was selected based on a systematic screen and analysis of the FDA-approved drug library and further biologic tests of the compounds. The researchers showed that CZ stimulated immunosuppressive functions of MSCs *in vitro*. In particular, CZ-pre-treated cells greatly inhibited T cell activation and proliferation which was demonstrated by a flow cytometry assay and CFSE living cell dye proliferation assay. Importantly, these beneficial effects were observed *in vivo* as well. Thus, when CZ-treated MSCs were injected intravenously into Thy1.1 antibody-induced AKI rats, a highly significant alleviation in inflammatory infiltration into renal tissue and fibrinoid necrosis of glomeruli was observed which was confirmed by PAS staining. Furthermore, the treatment was associated with reduced urine protein, and serum and urine creatinine indicating improvements in kidney functions. Beyond the downstream effects of CZ on MSCs, the authors could also determine to some extent the regulatory mechanisms by which that happened. It was found that CZ inhibited phosphorylation of transcription factor forkhead box O3 (FOXO3) independently from classical AKT or ERK signaling pathways. The latter could activate transcription of anti-inflammatory cytokines and IDO, which control the immunosuppressive functions of MSCs. However, as the authors themselves report, additional studies are needed to establish the exact molecular mechanism. It should also be mentioned that CZ appeared to be as good as interferon- γ (IFN-γ) and even superior to polyinosinic acid–polycytidylic acid [poly(I:C)] in terms of priming MSCs to anti-inflammatory phenotype. IFN-γ and poly(I:C) are the two compounds that are traditionally used in studies to switch MSCs to an anti-inflammatory subtype. Finally, it was demonstrated in this work that CZ did not impair the biological properties of MSCs such as differentiation, adhesion and expression of specific markers.

Another commonly used medication that was recently reported to have enhancing effects on the immunomodulatory functions of MSCs is metformin. In particular, research by Jang and colleagues has indicated efficiency of metformin, a standard drug for type 2 diabetes mellitus, in amplifying the immunosuppression of human AT-MSCs both *in vitro* and in mouse model of systemic lupus erythematosus (SLE) (178). First, the investigators showed that metformin-co-cultured MSCs increased the expression of anti-inflammatory enzymes and cytokines, namely, IDO, IL-10 and TGF-β and suppressed the proliferation of CD4+ T cells. Enhanced therapeutic effects of metformin-treated MSCs were also observed in Murphy Roths Large-lymphoproliferation strain mice, which tend to spontaneously develop SLE-like phenotype. Thus, mice that received once weekly intravenous injections of metformin-MCSs for 8 weeks, had a better disease course characterized by less severe glomerulonephritis, reduced proteinuria and decreased anti-dsDNA IgG antibody production. In addition, lower concentrations of CD90.2+CD4-CD8- double-negative T cells (most prevalent T cell subtype in SLE) and decreased Th17/Treg ratio were observed in various tissues of metformin-treated MSCs group compared to non-treated MSCs and the control group. Another important finding of the study is that metformin regulates the activities of AT-MSCs *via* the AMPK/mTOR/STAT1 signaling pathway. Specifically, metformin promoted phosphorylation of AMPK and STAT1 but reduced phosphorylation of mTOR, Raptor, Rictor, S6K and STAT3. It is important to note that previous studies also pointed out that the pleiotropic effects of metformin on cells such as immunomodulatory, anti-cancer and anti-aging activities are mediated by AMPK/mTOR signaling pathway (179–181). Furthermore, it was reported that the immunosuppressive properties of MSCs were controlled by STAT1 based on the observations that genetic knock-down and chemical inhibition of the protein resulted in a significant decrease in the expression of anti-inflammatory cytokines by the cells.

Besides medications, natural compounds such as caffeine, curcumin, melatonin and others have been reported to bolster the immunosuppressive capacity of MSCs. For example, caffeine-treated MSCs were shown to have a better immunomodulatory profile and improved therapeutic properties for the treatment of rheumatoid arthritis (182). The researchers first assessed the effects of caffeine on rat MSCs *in vitro*, in order to identify the most effective and safest concentration of the chemical. It was discovered that 0.5 mM is the most optimal concentration since it was associated with the highest levels of anti-inflammatory molecules (IDO, TGF-β, and IL-10) but the smallest amounts of pro-inflammatory cytokines (IFN-γ, IL-6, and IL-1β) without inhibitory effects on MSC proliferation in comparison to other concentrations. Importantly, higher concentrations of caffeine had significantly restrained proliferation of MSCs and abolished the secretion of some of the immunosuppressive molecules by the cells. The positive effects of 0.5 mM caffeine on MSCs were observed in Freund’s adjuvant-induced rat model of rheumatoid arthritis. Namely, a single intraperitoneal injection of caffeine-treated MSCs on day 14 of the disease resulted in an improvement of clinical and laboratory signs. That is, rats receiving caffeine-pulsed MSCs had a better mean arthritis index and gained more weight compared to MSCs alone and no MSCs groups. Moreover, the immunomodulatory effects of MSCs treated with caffeine were superior in comparison to ordinary MSCs. Specifically, caffeine-MSCs significantly reduced the secretion of C-reactive protein and rheumatoid factor as well as pro-inflammatory cytokines IL-1β, IFN-γ, TNF-α and IL-6 but elevated the levels of anti-inflammatory IL-10. Caffeine could stimulate the immunosuppression by MSCs through adenosine receptors on MSCs, actions on phosphodiesterases and direct interactions with neutrophils and macrophages (182, 183). Nevertheless, further research is needed to establish the exact signaling pathways and molecular events. Another natural compound that could be utilized for the immunomodulatory benefits of MSCs is curcumin, which was demonstrated in a recent work by Yang and colleagues (184). In this *in vitro* study, a 7-day treatment of human bone marrow-derived MSCs with 5 μmol/L curcumin resulted in a 6.65-fold increase in the expression of IDO1, which is one of the important immunoregulatory factors of MSCs. Similar to caffeine effects described above, curcumin actions on MSCs were also concentration-dependent. In particular, higher concentrations of this chemical appeared toxic to human bone marrow MSCs, namely, they induced apoptosis, inhibited cell proliferation, and reduced cell migration.

In addition to the aforementioned agents, some inorganic compounds have been reported to positively regulate the immunomodulatory attributes of MSCs. For instance, Yang and colleagues have recently shown that hydrogen sulfide gas (H2S) at a physiologic concentration was crucial for proper immunosuppressive functioning of gingiva-derived MSCs (GMSCs) (185). Thus, H2S-deficiency in GMSCs generated by siRNA knockdown of cystathionine β-synthase (CBS) and cystathionine γ-lyase (CSE) was associated with an impaired T cell migration, Tregs differentiation and Th17 cells differentiation as well as increased survival of T cells *in vitro*. Conversely, these impairments were repaired by NaHS treatment which restored the physiologic levels of H2S. The importance of H2S in regulation of GMSCs immunomodulatory capacity was further emphasized in a mouse model of acute colitis. Thus, H2S-deficient GMSCs could not mediate their therapeutic effects on diseased mice. In contrast, NaHS-treated GMSCs alleviated the severity of colitis, namely, weight loss, bloody diarrhea and loose stools. Furthermore, NaHS treatment of GMSCs re-established their immunosuppressive actions such as reduction of inflammation and Th17 polarization and promotion of Tregs. Importantly, this study is the first that investigated the role of hydrogen sulfide in regulation of immunomodulation by MSCs. Another inorganic compound that could boost the immunoregulatory properties of MSCs is cobalt (II) chloride (CoCl2). A study by Kwak and colleagues (186) indicated that CoCl2 could improve the anti-inflammatory qualities of human UC-MSCs for the treatment of asthma in a mouse model. First, the investigators showed that CoCl2-pretreated human UC-MSCs had a higher expression of anti-inflammatory mediator PGE2 and lower expression of the pro-inflammatory factors TNF-α and INF-γ *in vitro*. Moreover, treated human UC-MSCs suppressed proliferation and cluster formation of T cells when they were co-cultured with activated human peripheral blood mononuclear cells. Afterwards, the researchers determined the signaling pathway through which CoCl2 exerted its effects. Specifically, CoCl2 was found to mediate its effects *via* ERK-HIF-1α-miR-146a expression, i.e. CoCl2 induced expression of ERK which in turn stimulated HIF-1α production, and the latter caused enhanced miR-146a expression. MiR-146a upregulation then led to the anti-inflammatory effects described above. Lastly, CoCl2 was able to improve the immunomodulatory effects of hUC-MSCs in a mouse model of asthma induced by ovalbumin and polyinosinic-polycytidylic acid. Thus, CoCl2-pretreated hUC-MSCs injections into diseased animals were associated with reduced lung infiltration by macrophages, neutrophils and lymphocytes on day 24 of asthma, which was shown by bronchoalveolar lavage fluid and histological analyses. Taken together, drugs, natural compounds, inorganic chemicals and other miscellaneous agents have been reported to enhance the immunomodulatory capacity of MSCs. However, so far their use is limited to pre-clinical studies.

## 4 Clinical implementation and future prospectives

The promising results from preclinical studies of MSCs have spurred numerous attempts to utilize them in clinical trials. In 1995, MSCs therapy was assessed in humans for the first time and since then, the number of clinical studies evaluating it has been increasing exponentially (187). Recent reports indicate that there are more than 1,000 registered clinical trials using MSCs (187–189). The trials assess MSCs therapy for the treatment of autoimmune diseases and infectious diseases including COVID-19, as well as inflammatory and degenerative conditions of the heart, liver, lungs, skin, and nervous system. Importantly, the results of MSCs clinical research has been very encouraging. For instance, a recent systematic review and meta-analysis has demonstrated that MSCs could reduce mortality in patients with severe or critical COVID-19 (190). It is worthwhile to mention that numerous clinical trials have aimed to utilize immunomodulatory properties of MSCs. In these studies, the immunosuppressive qualities of MSCs were leveraged to treat Graft-versus-host disease, Crohn’s disease, multiple sclerosis, type 1 diabetes mellitus, gastrointestinal tumors, osteoarthritis, and atopic dermatitis (27, 191). With regards to the use of preconditioning and engineering strategies described in this paper, notably, almost all of the strategies mentioned in this review have reached clinical translation (192, 193). In particular, priming with cytokines and bioactive molecules, changing culture conditions, genetic engineering, and hypoxia preconditioning have been successfully utilized in clinical trials while autophagy regulation in MSCs is still awaiting its clinical application. For instance, in a Phase III trial (NCT01541579) the modification of culture conditions, and namely, the “rescue” of freshly thawed MSCs has been effective in enhancing their therapeutic potential for complex perianal fistulas in patients with Crohn’s disease who did not respond to conventional medications and biologicals (194). In another study (Phase II, NCT02017912), individuals with amyotrophic lateral sclerosis have been efficiently treated with MSCs primed with various trophic and growth factors (192). In two other clinical trials (Phase I/II, NCT01849159 and Phase II, NCT04042844), hypoxia-preconditioned MSCs are being evaluated for the treatment of severe pulmonary emphysema and chronic lumbar disc disease, respectively (195). In yet another study (Phase I/II, NCT02068794), genetically engineered MSCs to produce sodium iodine symporter are being assessed for the treatment of cancers of ovaries, fallopian tubes, and peritoneum (195). Given the wealth of positive preclinical results and ongoing clinical studies, it is expected that there will be more in-human trials on MSCs optimized with growth factors and cytokine pretreatment, hypoxia preconditioning, culture modification, and genetic engineering. With respect to autophagy regulation, which is also discussed in this review, there are quite limited preclinical data on this approach and this hampers its translation to clinical investigations.

## 5 Conclusion

MSCs have been considered as excellent therapeutic agents due to their ability to differentiate into diverse cell lineages and their capacity to regenerate and restore damaged tissues. However, compelling evidence suggests that the therapeutic effects of MSCs *in vivo* are largely produced by their immunomodulatory and trophic actions rather than differentiation and self-renewal. Specifically, numerous studies have demonstrated that MSCs could effectively suppress activation and proliferation of virtually every cell type of the immune system either through secretion of immunoregulatory factors or *via* direct contact with the cells of the immune system. The tremendous immunomodulatory potential of MSCs made them highly favorable candidates for the treatment of inflammatory and autoimmune conditions such as multiple sclerosis, myasthenia gravis, asthma, Crohn’s disease, rheumatoid arthritis and immune thrombocytopenia among many others in preclinical models and clinical trials. Nevertheless, the immunoregulatory effects of MSCs *in vivo* might be dramatically weakened due to poor engraftment rate and survival as well as hostile environment of the tissue. Furthermore, immunomodulatory properties of MSCs are characterized by plasticity, i.e. depending on the intensity of inflammation in the target tissue, MSCs produce varying immunoregulatory responses – in some cases MSCs can even enhance inflammation. Therefore, later studies have been focused on identifying strategies to enhance immunoregulatory abilities of MSCs.

Multiple approaches have been utilized so far to optimize the immunomodulatory functions of MSCs *in vivo*. They can be broadly classified into preconditioning strategies and genetic manipulations. Preconditioning of MSCs with pro-inflammatory cytokines such as TNF-α, IL-17 and IL-1β as well as treatment with immune receptor agonists like toll-like receptors agonists prior to transplantation could stimulate expression of immunomodulatory factors by MSCs and prepare them for the inflammatory environment of the target tissue. Similarly, varying culture conditions for MSCs, namely, changing confluency, using hypoxia and applying 3D spheroid cultures have been reported to induce expression of genes related to immunomodulation, cell survival, proliferation, migration and homing. Interestingly, preconditioning of MSCs with agents that control autophagy has also been shown to improve their immunosuppressive qualities, although the precise mechanisms of the process are yet to be elucidated. Finally, genetic manipulations to enhance expressions of genes regulating survival, proliferation and immunomodulation have also been leveraged to promote the therapeutic efficiency of MSCs.

## Author contributions

AS contributed conception and edited the manuscript. MS, YK, KR, BK, and VO wrote sections of the manuscript. All authors read and approved the submitted version.

## Funding

This research was funded by a Collaborative Research grant from Nazarbayev University (021220CRP0722).

## Conflict of interest

The authors declare that the research was conducted in the absence of any commercial or financial relationships that could be construed as a potential conflict of interest.

## Publisher’s note

All claims expressed in this article are solely those of the authors and do not necessarily represent those of their affiliated organizations, or those of the publisher, the editors and the reviewers. Any product that may be evaluated in this article, or claim that may be made by its manufacturer, is not guaranteed or endorsed by the publisher.
